# Iron Deficiency Anemia Following Bariatric Surgery: A 10-Year Prospective Observational Study

**DOI:** 10.3390/nu17020339

**Published:** 2025-01-18

**Authors:** Kinga Kędzierska, Marcin Dymkowski, Wiktoria Niegowska, Maria Humięcka, Ada Sawicka, Iwona Walczak, Zofia Maria Jędral, Michał Wąsowski, Agata Bogołowska-Stieblich, Artur Binda, Paweł Jaworski, Wiesław Tarnowski, Piotr Jankowski

**Affiliations:** 1 Department of Internal Medicine and Geriatric Cardiology, Centre of Postgraduate Medical Education, Orlowski Hospital, 00-416 Warsaw, Poland; 2 Department of General, Oncological and Bariatric Surgery, Medical Centre of Postgraduate Education, Orlowski Hospital, 00-416 Warsaw, Poland; 3 Department of Epidemiology and Health Promotion, School of Public Health, Center of Postgraduate Medical Education, 00-416 Warsaw, Poland

**Keywords:** bariatric, surgery, anemia, iron

## Abstract

Background: The long-term follow-up studies investigating the risk of anemia and iron deficiency following bariatric procedures are scarce. This study aimed to determine the influence of body weight reduction and type of bariatric surgery on iron metabolism parameters. Methods: We included 138 consecutive patients who underwent bariatric surgery (120 underwent sleeve gastrectomy and 18 underwent other types of bariatric surgery) between 2010 and 2016. At baseline and at follow-up (median observation: 10 years), examination weight and height were measured, and blood samples for iron metabolism parameters were taken. Results: Red blood cells (4.75 [4.59–4.96] 10^6^/μL vs. 4.51 [4.25–4.83] 10^6^/μL, *p* < 0.0001), hemoglobin (14.0 [13.3–14.7] g/dL vs. 13.0 [12.1–14.3] g/dL, *p* < 0.0001), and folic acid (7.4 [5.9–10.4] ng/ml vs. 6.0 [4.5–9.1] ng/mL, *p* = 0.01) were significantly lower, while anemia prevalence (6.52% vs. 28.99%, *p* < 0.0001) was significantly higher at the follow-up examination compared to the baseline values. In contrast, iron concentration (86.5 [68.0–109.0] µg/dL vs. 86.5 [55.0–110.0] µg/dL, *p* = 0.42) and TIBC values (351 [326–391] µg/dL vs. 345 [5311–387] µg/dL, *p* = 0.08) did not change significantly. The multivariable regression analyses showed that the only factors independently related to the hemoglobin concentration change were initial hemoglobin concentration, age, and bariatric procedures other than sleeve gastrectomy. Similarly, in the multivariable logistic analysis, the only variables independently related to the risk of anemia were age (adjusted odds ratio 0.93 [95% confidence intervals 0.89–0.97]), initial hemoglobin concentration (0.69 [0.49–0.97]), and procedures other than sleeve gastrectomy bariatric procedures (6.12 [1.86–20.15]). Conclusions: Age, initial hemoglobin concentration, and type of bariatric procedure but not sex, baseline iron serum level, or weight change are related to the risk of anemia in the long-term follow-up following bariatric surgery.

## 1. Introduction

Obesity is one of the pressing challenges of modern medicine. According to the WHO, in recent years, the population affected by obesity has nearly tripled and reached approximately 650 million adults [1]. In 2019, according to data from the Central Statistical Office, 56.6% of individuals in Poland aged 15 and above were overweight or obese, with 18.5% being obese [2]. As a result, the incidence of obesity-related diseases such as diabetes mellitus type 2, hypertension, dyslipidemia, osteoarthritis, obstructive sleep apnea, and some neoplasms is also increasing. The increase in the prevalence of obesity is also evident among patients with obesity-related complications [3]. This makes obesity a significant health priority. Modern methods of treating obesity include lifestyle modification with dietary restrictions, increased physical activity, pharmacological treatment, and surgical intervention.

Among many surgical methods, the most frequently performed are sleeve gastrectomy (SG), Roux-en-Y gastric bypass (RYGB), and mini gastric bypass (MGB) [4,5]. The procedure of SG is usually performed laparoscopically and removes about 80% of the stomach along the greater curvature. As it is not associated with the interruption in the continuity of the GI tract, it does not require any anastomoses. SG has a low rate of complications. On the other hand, RYGB combines the reduction in gastric volume and a bypass of the proximal small bowel and stomach except for a small part of the gastric cardia (50–100 cm). As it requires the disruption of GI tract continuity, two anastomoses are required to form a Y-shaped bowel connection. The MGB procedure, similar to RYGB, reduces the stomach volume and bypasses the small bowel. The gastric pouch is made of cardia and a part of the small curvature, and the bypassed proximal part of the GI tract is longer (150–200 cm). Moreover, just one anastomosis is required for the continuity restoration of the GI tract.

A common complication of obesity is iron deficiency (ID) leading to iron deficiency anemia. The key factor leading to ID in individuals with obesity is inflammation, which affects the uptake, storage, and transportation of iron. Obesity is associated with chronic low-grade inflammation, inducing an increase in pro-inflammatory cytokines, including IL-6, which is responsible for the apoptosis promotion of immature erythroblasts and hepcidin overproduction [6]. Hepcidin is a positive acute-phase protein that reduces intestinal iron absorption and erythropoiesis. Obesity-related inflammation decreases after bariatric surgery, leading to reduced hepcidin serum levels and increased iron absorption [7]. However, bariatric surgery may lead to malabsorption, resulting in iron deficiency or exacerbation of the ID that was already present before the surgery.

As the issue of ID associated with obesity and bariatric surgery is complex, other factors need to be considered, including sex, age, type of surgery, and comorbidities [5,8]. It has been admittedly proven that female sex and RYGB surgery are associated with an increased risk of ID development following bariatric surgery. However, the current literature is inconsistent regarding the role of age in the pathogenesis of ID development after bariatric procedures. Furthermore, the extent of ID prevalence after bariatric surgery varies between studies from 18% to 54%, while the prevalence of anemia ranges from 6% to 64% [9]. Additionally, weight loss may be associated with an increased risk of anemia [10], potentially due to caloric restriction leading to insufficient intake of essential micronutrients, as well as impairments in nutrient absorption mechanisms.

Moreover, there are few studies involving long-year follow-up that enable the observation of long-term complications and iron metabolism parameters after bariatric surgery. Therefore, we conducted a study involving a ten-year follow-up after bariatric surgery to determine the influence of body weight reduction, type of bariatric surgery, and other factors on iron metabolism parameters.

## 2. Subjects and Methods

Consecutive patients undergoing bariatric surgery between 2010 and 2016 were included and participated in the follow-up examination in 2022–2023. Qualification criteria for bariatric surgery were based on the clinical practice guidelines of the European Association for Endoscopic Surgery (EAES) on bariatric surgery and included patients with BMI ≥ 40 kg/m^2^, patients with BMI ≥ 35–40 kg/m^2^ with associated comorbidities that are expected to improve with weight loss, and patients with BMI ≥ 30–35 kg/m^2^ and type 2 diabetes and/or arterial hypertension with poor control despite optimal medical therapy [11]. The only exclusion criterion was the lack of consent to participate in the study. Trained research staff collected data using standardized methods. They reviewed patient medical notes and interviewed and examined the patients using the standardized data collection forms. At baseline and at the follow-up examination, data on demographic characteristics, personal medical history, anthropometric measurements, and blood samples for blood cell count, iron, B_12_, folic acid levels, and total iron-binding capacity (TIBC) were taken. The concentrations of vitamin B12 and folic acid refer to serum levels.

The assays and reagents utilized in this research included the following: complete blood count (CBC) was performed using automated hematological analysis with the direct current (DC) method and sheath flow in the RBC channel. Hemoglobin levels were determined using the sodium lauryl sulfate (SLS) method. The lipid profile was evaluated through enzymatic spectrophotometry. Serum iron concentrations were measured using a spectrophotometric method with ferrozine. Vitamin B12 and folic acid levels were analyzed using a chemiluminescent immunoassay (CLIA). Ferritin levels were quantified using an electrochemiluminescence immunoassay (ECLIA).

In addition, during the follow-up examination, the ferritin and transferrin levels, as well as transferrin saturation, were measured. Obesity has been defined as body mass index (BMI) ≥ 30 kg/m^2^ [1]. Comorbidities have been defined according to the guidelines valid at the time of the baseline examination. Anemia was defined as hemoglobin < 13 g/dL in men and <12 g/dL in women [12]. The used norm for TIBC values was >450 µg/dL [13]. Iron deficiency was defined as serum iron levels below 90 µg/dL in men and below 60 µg/dL in women.

All participants gave written informed consent. The Bioethics Committee of the Centre of Postgraduate Medical Education (Warsaw, Poland) approved the study protocol (no. 16/2022, 16 February 2022).

### Statistical Analysis

Categorical variables were reported as percentages and continuous variables as medians with first and third quartiles or means ± standard deviation. The Pearson χ^2^ test was applied to all categorical variables. The Shapiro–Wilk test was used to assess the normality of continuous variables. Normally distributed continuous variables were compared using Student’s *t*-test for dependent or independent variables, as appropriate, while the variables without normal distributions were evaluated using the Mann–Whitney U test or the Wilcoxon test. A two-tailed *p*-value of less than 0.05 indicated statistical significance. Subsequently, multivariable backward stepwise linear analysis using the general linear model is implanted in the Statistica package and the multivariable logistic analysis was performed. The baseline model in all multivariable analyses included age, sex, BMI, type of surgery, concentrations of total cholesterol, triglycerides, vitamin B_12_, folic acid, iron, Hb, TIBC values, and the presence of hypertension, diabetes, and dyslipidemia. The power analysis was conducted to determine the minimum sample size required to detect the difference in the proportion of patients with iron deficiency between the baseline and follow-up examinations. The required sample size to achieve 80% power for detecting a difference of 15 percentage points at a significance criterion of α = 0.05 was 135. The statistics were calculated using STATISTICA 13 software (TIBCO Software, Palo Alto, CA, USA).

## 3. Results

We recruited and studied 154 consecutive patients. We excluded 16 cases from the analyses because of missing key variables (iron level, TIBC) at baseline or follow-up. The analyzed and not analyzed patients did not differ significantly in terms of age, sex, and BMI. The study group characteristics are presented in Table 1. The only significant sex-based difference was in dyslipidemia proportions (Table 1). The median BMI was 42.21 (38.22–46.60) kg/m^2^. All study participants had BMI ≥ 30.0 kg/m^2^; a BMI of at least 35.0 kg/m^2^ was observed in 92.02% of the study participants, at least 40.0 kg/m^2^ in 68.12%, while it was at least 50.0 kg/m^2^ in 15.22%. The baseline iron metabolism parameters are shown in Table 2.

A total of 120 patients (86.96%) underwent SG as the first bariatric procedure, while 18 patients (13.04%) underwent other types of bariatric surgery as the first bariatric procedure, including RYGB (*N* = 5), MGB (*N* = 10), adjustable gastric band (AGB, *N* = 1), or intragastric balloon placement (*N* = 2). No patient underwent single anastomosis sleeve ileal bypass (SASI). The SG group differed significantly from the other bariatric procedures group in terms of age (43.06 ± 9.71 years vs. 48.06 ± 8.65 years, *p* = 0.041) and BMI (43.05 kg/m^2^ [39.93 kg/m^2^–47.12 kg/m^2^] vs. 37.25 kg/m^2^ [34.00 kg/m^2^–41.47 kg/m^2^], *p* = 0.0003). There was no statistically significant difference in sex distribution between the groups (76.67% women vs. 61.11% women, *p* = 0.1571). In the SG group, 16 (13.33%) patients had a second bariatric procedure, of which 12 patients had MGB, three had RYGB, and one had SASI. In the other bariatric procedures group, three (16.67%) patients had a second bariatric procedure, all of which were SG.

The median observation period was 10 years [10 years–11 years]. The observation period was similar in females and males (10 [10,11] years vs. 10 [9,10,11] years, *p* = 0.51), as well as in the SG group and the other bariatric procedures group (10 [10,11,12] years vs. 10 [9,10] years, *p* = 0.077).

The median weight at follow-up was 94.5 (85.0–112) kg in the whole studied group, 100.0 (85.0–114.0) kg in females and 90.0 (84.0–101.0) kg in males (*p* = 0.11). The mean weight change was −21.48 ± 16.03 kg, while the median BMI change was −6.7 kg (−10.6 kg–−3.8 kg), Table 1. A BMI below 25.0 kg/m^2^ at the follow-up examination was found in 5.8% of patients, 28.0% were overweight, 26.8% had a BMI in the range of 30.0–35.0 kg/m^2^, 25.4% in the range of 35.0–40.0 kg/m^2^, and 23.2% had a BMI of at least 40 kg/m^2^. The medians of weight change in the SG group (−19.8 kg [−30.5 kg–−11.5 kg]) and in the other bariatric procedures group (−21.1 kg [−29.8 kg–−13.5 kg]) were similar (*p* = 0.44). Similarly, we did not find any significant difference in the change in BMI between the groups mentioned above (6.7 kg/m^2^ [−10.3 kg/m^2^–−3.6 kg/m^2^] vs. −6.8 kg/m^2^ [−10.7 kg/m^2^–−4.5 kg/m^2^], *p* = 0.56).

**Figure 1:**
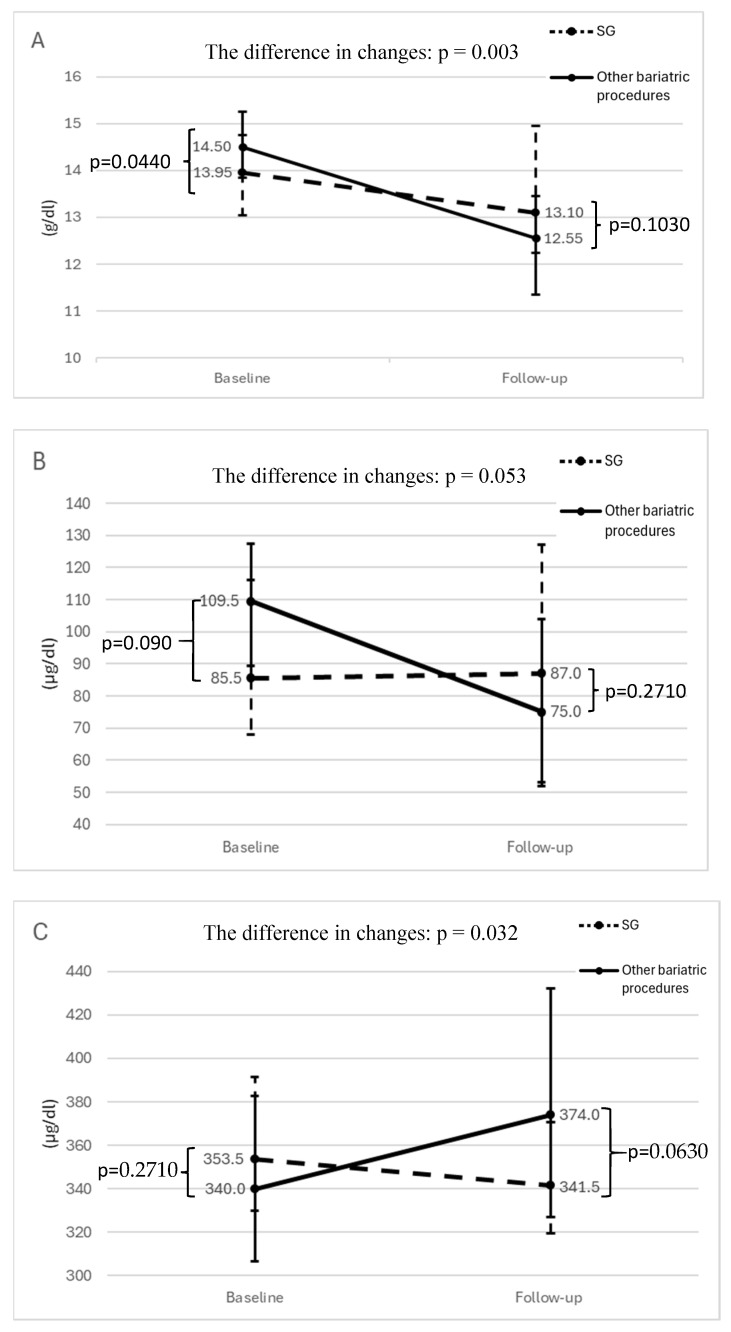
The change in hemoglobin concentration (**A**), iron concentration (**B**), and TIBC value (**C**) by the type of surgery. Dots are medians, whiskers are interquartile ranges. “Baseline” refers to the values prior to bariatric surgery, while “Follow-up” refers to the values 10 years after the surgery.

During the observation period, the number of red blood cells and the hemoglobin concentration significantly decreased (Table 2). We found significant changes when we analyzed females, men, the SG group, and the other bariatric procedures group separately (Table 2 and Table 3). The reduction in the hemoglobin level was significantly higher in the other bariatric procedures group than in the SG group (−2.15 [−3.40–1.00] g/dL vs. −0.60 [−1.65–0.20] g/dL, *p* < 0.01), Figure 1A. The type of bariatric surgery, along with age and initial hemoglobin concentration, were the only independent predictors of change in the hemoglobin level other than the observation period (Table 4A) (Figure 1A).

On the other hand, we did not find any significant change in the iron level in either of the studied groups. However, the difference in changes between the surgical groups was of borderline significance (0.08 ± 46.22 µg/dL vs. −23.94 ± 63.86 µg/dL, *p* = 0.05), Figure 1B. In the multivariate regression analysis, the type of bariatric surgery, age, and folic acid concentration were the only independent predictors of change in the iron concentration over the observation period (Table 4A). Although we did not detect any significant change over time in the TIBC, the difference in TIBC change between the surgical groups was statistically significant (−13.0 [−55.0–29.0] µg/dL vs. 25.5 [−27.0–64.0] µg/dL, *p* = 0.03) (Figure 1C). Initial hemoglobin concentration and the type of bariatric surgery were proved to be independent predictors of change in the TIBC values over the observation period (Table 4A) (Figure 1B,C).

At the follow-up examination, total transferrin concentration was 284.5 [253.0–338.0] µg/dL (females 295.0 [263.0–338.0] µg/dL vs. males 253.0 [227.0–327.0] µg/dL, *p* = 0.016) and (SG 281.0 [253.0–327.0] µg/dL vs. other 327.0 [263.0–357.0] µg/dL, *p* = 0.0797). Baseline total transferrin saturation was 24.5 [16.35–32.95] %, (females 24.3 [14.8–32.8] % vs. males 26.1 [22.7–33.5] %, *p* = 0.2297), and (SG 24.7 [17.0–32.8] % vs. other 23.1 [8.6–33.3] %, *p* = 0.2735). Initial total ferritin concentration was 28.5 [10.6–67.5] µg/L, (females 25.8 [10.0–67.7] µg/L vs. males 37.1 [12.1–59.1] µg/L *p* = 0.446) and (SG 33.5 [12.1–73.7] µg/L vs. other 11.4 [8.7–37.1] µg/L, *p* = 0.0252).

**Figure 2:**
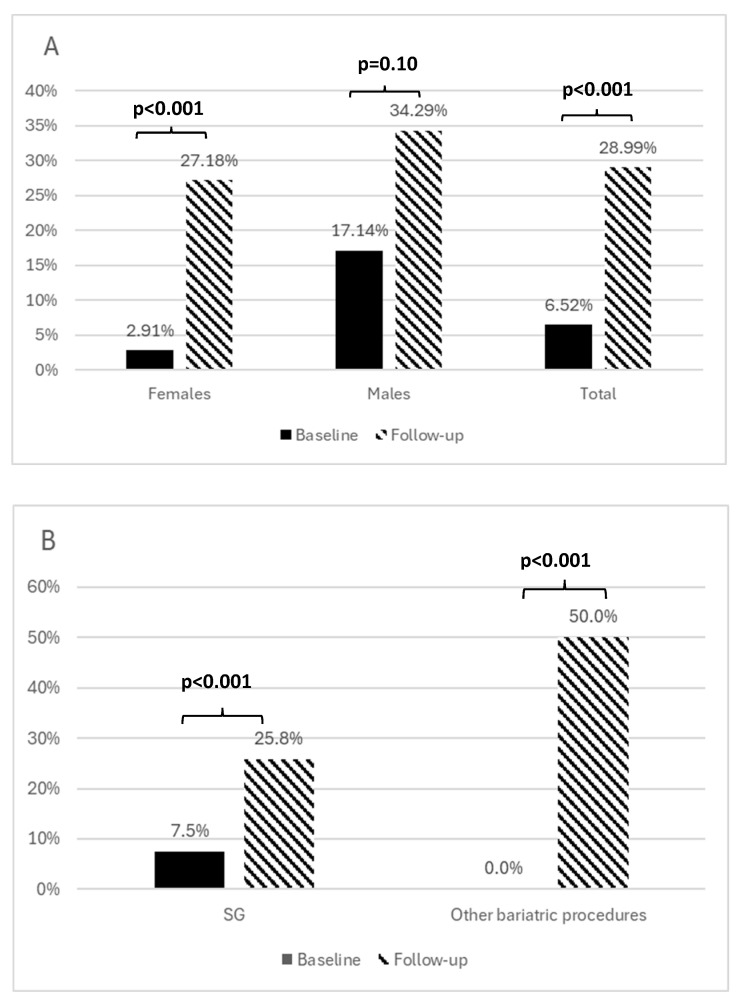
The prevalence of anemia before and after the surgery by sex (**A**) and type of bariatric procedure (**B**).

The prevalence of anemia increased during the observation period, as shown in Figure 2 (Figure 2A,B). The multivariate logistic analysis showed that the type of bariatric surgery, along with age and initial hemoglobin concentration, were the only independent predictors of change in the prevalence of anemia over the observation period (Table 4B).

## 4. Discussion

Our study investigated the influence of multiple factors on iron homeostasis parameters following bariatric surgery. We observed statistically significant changes in hemoglobin levels and the proportion of patients with iron deficiency during the long-term follow-up. When interpreting these findings, it is important to consider the significant discrepancies in group sizes. Notably, we did not observe a significant association between baseline weight or weight change and the iron metabolism parameters or the risk of anemia in the long-term follow-up. We also did not detect a significant association between sex and changes in iron metabolism parameters.

We observed a statistically significant reduction in hemoglobin levels following surgery. However, there was no statistically significant difference in median iron levels before and after the procedure. It is important to note that median values for both parameters (hemoglobin and iron) remained within the normal range.

In our study, we observed an association between baseline hemoglobin levels and the occurrence of post-bariatric surgery anemia in patients. This correlation is likely attributed to diminished initial iron reserves, which may predispose individuals to anemia before complete adaptation to the metabolic changes postoperatively. A plethora of studies in the field substantiate these findings [14,15].

Regarding the prevalence of anemia, it is notable that the incidence was higher among patients who had undergone bariatric procedures other than SG as their first surgery. It is crucial to consider the significant discrepancies in group sizes when interpreting these findings. The decrease in median hemoglobin and iron levels was significantly higher in the other bariatric surgery group. This highlights the importance of monitoring iron metabolism to pre-empt potential complications over time.

Interestingly, in studies with shorter follow-up periods after bariatric surgery, a higher risk of iron deficiency anemia was noted after 2 years post-operation. The need for intravenous iron supplementation increased after 3 years post-surgery [16]. However, after 10 years, the percentage of patients experiencing this condition decreased. This may indicate that iron deficiency anemia was more prevalent in the early years following surgery [17]. However, this study lacks a comprehensive comparison with other types of surgeries. Only Roux-en-Y gastric bypass procedures are presented in the cited research. Analyzing other studies [18], an improvement in iron metabolism can be observed within months of the surgical procedure.

It is worth noting that many studies have observed sleeve gastrectomy (SG) to pose a lower risk of iron deficiency anemia for patients. An alternative trend has been identified in the cited study [19]. Iron levels were higher in patients with gastric bypass (GB) compared to SG patients after a short observation period. Notably, in this study, the lost body mass was also greater after GB than after SG, which may indicate other factors influencing iron metabolism.

Furthermore, no correlation was identified between weight loss and the incidence of anemia or iron deficiency incidence, as confirmed in recent studies [10,20]. These results indicate that the surgical procedure exerts a more profound influence on these outcomes than weight reduction.

Across the entire study cohort, no notable change in TIBC was detected over time. Nonetheless, a statistically significant discrepancy in TIBC alterations emerged between the distinct surgical groups. The decrease in TIBC may also raise concerns regarding anemia related to chronic diseases. Regrettably, we lacked studies to substantiate this hypothesis. For a thorough examination, forthcoming investigations should incorporate inflammatory biomarkers, including CRP, interleukin-6, or alpha-1 acid glycoprotein. Surgery-related inflammation may enhance iron absorption by upregulating divalent metal transporter 1 (DMT1) expression. Consequently, systematic iron supplementation may not be imperative in the initial year post-sleeve gastrectomy, although long-term monitoring remains prudent. Additionally, inflammation directly impacts iron absorption in the intestines by modulating the expression of iron transporters DMT1 and ferroportin [14]. Although previous studies indicated that iron deficiency is more likely a long-term consequence of bariatric surgery rather than an immediate effect, our study suggests a different trend. Special attention should be paid to the pathogenesis of obesity, which may involve inflammation and genetic predispositions. This issue is of considerable clinical relevance, given that patients with obesity, and especially those undergoing bariatric surgery, exhibit impaired iron absorption mechanisms [21]. Furthermore, the efficacy and standardization of iron supplementation protocols remain inconsistent across studies, underscoring the need for further investigation.

We found no significant relationship between sex and the risk of anemia over the long-term observation following bariatric surgery. This finding is particularly unexpected in light of the prevailing literature suggesting a female predisposition to anemia [10,22]. The difference in the age of the studied patients could potentially explain the different findings. It also has been observed that iron status markers showed no statistically significant differences pre- and post-operatively. The results of these studies align with our findings, which may indicate that undergoing surgery at an older age confers protection against iron depletion in women. This is further supported by the study’s findings, which noted that patients with iron deficiency were younger than those who never experienced iron deficiency [15].

Furthermore, we observed that age exerts a protective effect on hemoglobin and iron levels. This phenomenon may be associated with a decreased prevalence of menstruating female patients, further confirming the lack of correlation between anemia and sex. Importantly, studies indicate that bariatric surgery effectively restores menstrual regularity in obese women of reproductive age, which is correlated with weight loss [23,24]. It should be noted that iron demand diminishes with advancing age, which could contribute to these outcomes.

Recent studies challenge the necessity of blanket high-dose supplementation, a notion congruent with our findings. We found no statistically significant association between hemoglobin concentration and vitamin B12 levels, supporting findings from cohort studies challenging the necessity of high-dose prophylactic multivitamin prescriptions. Nonetheless, we identified a reduction in folic acid levels following bariatric surgery. This could be attributed to the tendency of older individuals to exhibit decreased folate absorption, or it could represent a complication of the surgery. Crucially, results of the multivariable analysis highlight the fact that elevated folate levels serve as a protective factor against diminished iron stores and subsequent iron deficiency. This relationship may be correlated with dietary behaviors and optimal nutritional status. Notably, the predictive factor pertains solely to iron deficiency and does not impact hemoglobin concentrations, thus reinforcing this hypothesis. These aspects warrant consideration in future research, focusing on verifying these issues among younger demographics. While iron supplementation should be administered as needed, routine high doses may be superfluous. The close monitoring of patients is imperative, and supplementation should be tailored to individual needs [25].

Limitations of our study include its single-center design, limited sample size, and the absence of preoperative ferritin and transferrin measurements. We also did not analyze the morphological parameters of blood cells. Additionally, we did not use the gold standard to assess iron deficiency, which is microscopy of a bone marrow sample stained with Perls’ stain [26]. Furthermore, the uneven distribution of patients across different surgical groups and sexes affects the generalizability of our findings. However, it should be underlined that the difference in the number of surgical groups mirrors the contemporary everyday reality. Future investigations should explore the cumulative ramifications of various bariatric surgery procedures. Furthermore, factors such as vitamin and mineral supplementation, including iron, dietary habits, and lifestyle, were not assessed or controlled, which may have influenced iron metabolism and the occurrence of anemia among these patient groups. Additionally, we did not divide the female groups into menstruating and non-menstruating, which could also influence the final results. Our study participants underwent several kinds of bariatric procedures. However, no one underwent the single anastomosis sleeve ileal bypass at baseline [27]. Furthermore, our patient group only included individuals of Caucasian race. Therefore, our results should not be directly applied to the other populations. On the other hand, we included a consecutive unselected cohort of patients. Therefore, this analysis provides reliable information on the prevalence of anemia in the long-term follow-up following bariatric surgery.

## 5. Conclusions

We found that age and the type of bariatric procedure are significantly related to the risk of anemia following bariatric treatment. Individuals who undergo bariatric procedures other than sleeve gastrectomy have a higher predisposition to anemia. We did not find evidence of an independent association between sex, baseline iron metabolism parameters, weight change, and the risk of anemia in the long-term follow-up bariatric surgery. These results should be considered when qualifying patients for bariatric surgery and can be seen as an argument in favor of sleeve gastrectomy.

## Figures and Tables

**Table 1:** The study group characteristics.

	All (n = 138)	Females (n = 103)	Males (n = 35)	*p*-Value
Baseline				
Age, mean (SD)	43.71 ± 9.69	43.08 ± 10.17	45.57 ± 7.98	0.1813
Weight (kg), mean (SD)	120.32 ± 19.05	121.15 ± 17.95	117.89 ± 22.09	0.2130
Height (m), mean (SD)	1.67 ± 0.08	1.67 ± 0.08	1.66 ± 0.08	0.7726
BMI (kg/m^2^), median (ICR)	42.21 (38.22–46.60)	43.03 (38.85–46.60)	40.60 (36.17–47.40)	0.1360
Hypertension, n (%)	91 (65.94)	64 (62.14)	27 (77.14)	0.0529
Diabetes, n (%)	42 (30.43)	28 (27.18)	14 (40.00)	0.0772
Dyslipidemia, n (%)	32 (23.18)	20 (19.42)	12 (34.29)	0.0359
Total cholesterol (mg/dL), median (ICR *)	189.5 (170.0–213.0)	195.0 (169.0–216.0)	189.0 (175.0–212.0)	0.8505
Triglycerides (mg/dL), median (ICR *)	137.0 (100.0–180.0)	133.0 (100.0–180.0)	138.0 (96.0–195.0)	0.9649
Type of surgery				
Sleeve gastrectomy, n (%)	120 (86.96)	92 (89.32)	28 (80.00)	0.1570
Other bariatric procedures, n (%)	18 (13.04)	11 (10.68)	7 (20.00)	0.0990
Follow-up				
Weight (kg), median (ICR *)	94.5 (85.0–112)	100.0 (85.0–114.0)	90.0 (84.0–101.0)	0.1100
Weight change (kg), mean (SD)	−21.48 ± 16.03	−20.90 ± 16.65	−23.20 ± 14.14	0.4640
BMI (kg/m^2^), median (ICR *)	34.82 (30.06–39.51)	35.78 (30.47–40.06)	33.87 (28.96–36.63)	0.0976
BMI change (kg/m^2^), median (ICR *)	−6.65 (−10.64–−3.78)	−6.54 (−10.86–−3.59)	−7.34 (−10.27–−4.78)	0.5030

* ICR—interquartile range.

**Table 2:** Blood cell count and iron metabolism parameters before and after surgery.

	Baseline	Follow-Up	*p*-Value
All patients, *N* = 138			
Red blood cells (10^6^/μL), median (ICR *)	4.75 (4.59–4.96)	4.51 (4.25–4.83)	<0.0001
Hemoglobin (g/dL), median (ICR *)	14.0 (13.3–14.7)	13.0 (12.1–14.3)	<0.0001
Iron (μg/dL), median (ICR *)	86.5 (68.0–109.0)	86.5 (55.0–110.0)	0.4217
TIBC (μg/dL), median (ICR *)	351.0 (326.0–391.0)	345.0 (311.0–387.0)	0.0816
B12 (pg/mL), median (ICR *)	305.5 (251.0–400.0)	337.5 (262.0–434.0)	0.6600
Folic acid (ng/mL), median (ICR *)	7.4 (5.9–10.4)	6.0 (4.5–9.1)	0.0107
Iron deficiency, n (%)	37 (26.81)	47 (34.06)	0.0953
TIBC above normal, n (%)	13 (9.42)	7 (5.07)	0.0817
Females, *N* = 103			
Red blood cells (10^6^/μL), median (ICR *)	4.75 (4.59–4.94)	4.51 (4.25–4.83)	<0.0001
Hemoglobin (g/dL), median (ICR *)	14.0 (13.3–14.7)	12.9 (11.7–14.3)	<0.0001
Iron (μg/dL), median (ICR *)	86 (68–109)	84 (50–110)	0.3207
TIBC (μg/dL), median (ICR *)	353 (333–392)	350 (322–295)	0.3330
B12 (pg/mL), median (ICR *)	302 (248–400)	329 (252–415)	0.9908
Folic acid (ng/mL), median (ICR *)	7.3 (5.88–10.2)	6.0 (4.5–8.9)	0.0249
Iron deficiency, n (%)	17 (16.50)	31 (30.10)	0.0105
TIBC above normal, n (%)	9 (8.73)	5 (4.85)	0.1342
Males, *N* = 35			
Red blood cells (10^6^/μL), median (ICR *)	4.73 (4.52–5.11)	4.52 (4.36–4.84)	0.0011
Hemoglobin (g/dL), median (ICR *)	14.1 (13.2–14.8)	13.4 (12.4–14.3)	0.0147
Iron (μg/dL), median (ICR *)	87 (65–117)	90 (69–110)	0.9543
TIBC (μg/dL), median (ICR *)	339 (317–388)	328 (293–371)	0.0840
B_12_ (pg/mL), median (ICR *)	341 (356–408)	365 (285–453)	0.3463
Folic acid (ng/mL), median (ICR *)	8.3 (6.2–11.8)	5.9 (4.6–12.5)	0.2416
Iron deficiency, n (%)	20 (57.14)	16 (45.71)	0.1694
TIBC above normal, n (%)	4 (11.42)	2 (5.71)	0.1967

* ICR—interquartile range.

**Table 3:** Blood morphological parameters and iron metabolism parameters by type of bariatric surgery.

	Baseline	Follow-Up	*p*-Value
Sleeve gastrectomy			
Red blood cells (10^6^/μL), median (ICR *)	4.75 [4.58–4.95]	4.53 [4.25–48.4]	<0.0001
Hemoglobin (g/dL), median (ICR *)	13.95 (13.2–14.6)	13.1 (12.2–14.3)	<0.0001
TIBC (μg/dL), median (ICR *)	353.5 (330.0–391.5)	341.5 (308.0–384.0)	0.0154
Iron (μg/dL), median (ICR *)	85.5 (67.5–105.5)	87.0 (58.0–110.0)	0.9778
Other bariatric procedures			
Red blood cells (10^6^/μL), median (ICR *)	4.90 [4.62–5.12]	4.45 [4.26–4.65]	0.0014
Hemoglobin (g/dL), median (ICR *)	14.5 (13.7–15.4)	12.6 (10.7–13.4)	0.0007
TIBC (μg/dL), median (ICR *)	340.0 (318.0–369.0)	374.0 (327.0–432.0)	0.2485
Iron (μg/dL), median (ICR *)	109.5 (79.0–127.0)	75.0 (35.0–109.0)	0.1387

* ICR—interquartile range.

**Table 4:** Variables independently related to the change in the hemoglobin concentration, iron concentration and TIBC values (A), and prevalence of anemia (B) over the observation period.

(A)		
Variable	Regression Coefficient ± Standard Error	*p*-Value
Change in hemoglobin concentration		
Initial hemoglobin concentration	−0.3810 ± 0.0751	<0.0010
Type of surgery; sleeve gastrectomy—0, other bariatric procedures—1	−0.2520 ± 0.0755	0.0010
Age	0.3090 ± 0.0753	<0.0010
Change in iron concentration		
Type of surgery; sleeve gastrectomy—0, other bariatric procedures—1	−0.2220 ± 0.0840	0.0090
Age	0.1920 ± 0.0840	0.0236
Folic acid concentration	0.1670 ± 0.0836	0.0483
Change in TIBC values		
Initial hemoglobin concentration	0.1760 ± 0.0840	0.0380
Type of surgery; sleeve gastrectomy—0, other bariatric procedures—1	0.1770 ± 0.0840	0.0365
**(B)**		
**Variable**	**Adjusted Odds Ratio** **(95% Confidence Intervals)**	***p*-Value**
Initial hemoglobin concentration	0.69 (0.49–0.97)	0.0326
Type of surgery; sleeve gastrectomy—0, other bariatric procedures—1	6.12 (1.86–20.15)	0.0031
Age, per one year	0.93 (0.89–0.97)	0.0025

## Data Availability

The datasets generated during and analyzed during the current study are available from the corresponding author upon reasonable request due to protect participant privacy.
